# A human monoclonal antibody neutralizing SARS-CoV-2 Omicron variants containing the L452R mutation

**DOI:** 10.1128/jvi.01223-24

**Published:** 2024-11-04

**Authors:** Saskia C. Stein, Guido Hansen, George Ssebyatika, Luisa J. Ströh, Okechukwu Ochulor, Elisabeth Herold, Britta Schwarzloh, Doris Mutschall, Jasmin Zischke, Anne K. Cordes, Talia Schneider, Imke Hinrichs, Rainer Blasczyk, Hannah Kleine-Weber, Markus Hoffmann, Florian Klein, Franziska K. Kaiser, Mariana Gonzalez-Hernandez, Federico Armando, Malgorzata Ciurkiewicz, Georg Beythien, Stefan Pöhlmann, Wolfgang Baumgärtner, Albert Osterhaus, Thomas F. Schulz, Thomas Krey

**Affiliations:** 1 Institute of Virology, Hannover Medical School, Hannover, Germany; 2 Institute of Biochemistry, Center of Structural and Cell Biology in Medicine, University of Lübeck, Lübeck, Germany; 3 Laboratory of Experimental Immunology, Institute of Virology, University of Cologne, Cologne, Germany; 4 Institute of Transfusion Medicine and Transplant Engineering, Hannover Medical School, Hannover, Germany; 5 German Primate Center, Leibniz Institute for Primate Research, and Faculty of Biology and Psychology, University Göttingen, Göttingen, Germany; 6 German Center for Infection Research, Partner Site Bonn-Cologne, Cologne, Germany; 7 Center for Molecular Medicine Cologne (CMMC), University of Cologne, Cologne, Germany; 8 Research Center for Emerging Infections and Zoonoses, University of Veterinary Medicine Hannover, Foundation, Hannover, Germany; 9 Department of Pathology, University of Veterinary Medicine Hannover, Foundation, Hannover, Germany; 10 Excellence Cluster 2155 RESIST, Hannover, Germany; 11 Global Virus Network, Center of Excellence, University of Veterinary Medicine, Hannover, Germany; 12 German Center for Infection Research, Partner Site Hannover-Braunschweig, Hannover, Germany; 13 German Center for Infection Research, Partner Site Hamburg-Lübeck-Borstel-Riems, Hannover, Germany; 14 Centre for Structural Systems Biology, Hamburg, Germany; St. Jude Children's Research Hospital, Memphis, Tennessee, USA

**Keywords:** SARS-CoV-2, Omicron variant, neutralizing antibody, neutralization escape

## Abstract

The effectiveness of SARS-CoV-2 therapeutic antibodies targeting the spike (S) receptor-binding domain (RBD) has been hampered by the emergence of variants of concern (VOCs), which have acquired mutations to escape neutralizing antibodies (nAbs). These mutations are not evenly distributed on the RBD surface but cluster on several distinct surfaces, suggesting an influence of the targeted epitope on the capacity to neutralize a broad range of VOCs. Here, we identified a potent nAb from convalescent patients targeting the receptor-binding domain of a broad range of SARS-CoV-2 VOCs. Except for the Lambda and BA.2.86 variants, this nAb efficiently inhibited the entry of most tested VOCs, including Omicron subvariants BA.1, BA.2, XBB.1.5, and EG.5.1 and to a limited extent also BA.4/5, BA.4.6, and BQ.1.1. It bound recombinant S protein with picomolar affinity, reduced the viral load in the lung of infected hamsters, and prevented the severe lung pathology typical for SARS-CoV-2 infections. An X-ray structure of the nAb-RBD complex revealed an epitope that does not fall into any of the conventional classes and provided insights into its broad neutralization properties. Our findings highlight a conserved epitope within the SARS-CoV-2 RBD that should be preferably targeted by therapeutic antibodies and inform rational vaccine development.

**IMPORTANCE:**

Therapeutic antibodies are effective in preventing severe disease from SARS-CoV-2 infection and constitute an important option in pandemic preparedness, but mutations within the S protein of virus variants (e.g., a mutation of L452) confer resistance to many of such antibodies. Here, we identify a human antibody targeting the S protein receptor-binding domain (RBD) with an elevated escape barrier and characterize its interaction with the RBD functionally and structurally at the atomic level. A direct comparison with reported antibodies targeting the same epitope illustrates important differences in the interface, providing insights into the breadth of antibody binding. These findings highlight the relevance of an extended neutralization profiling in combination with biochemical and structural characterization of the antibody-RBD interaction for the selection of future therapeutic antibodies, which may accelerate the control of potential future pandemics.

## INTRODUCTION

Broadly neutralizing monoclonal antibodies (bnAbs) targeting the SARS-CoV-2 spike protein (S) inhibit SARS-CoV-2 infection both in cell culture and in the infected host. Many laboratories have therefore isolated human nAbs from COVID-19 patients, convalescents, or individuals vaccinated against SARS-CoV-2 (1–8). Some antibodies have received U.S. Food and Drug Administration (FDA) and/or European Medicines Agency (EMA) approval for clinical use either alone (bamlanivimab/ LY-CoV555) or in combination with a second antibody, for example, bamlanivimab (LY-CoV555) with etesevimab (LY-CoV016) or casirivimab (REGN10933) with imdevimab (REGN10987) [(9); clinical trial data reviewed in (10)]. Potently neutralizing human monoclonal antibodies can provide a clinical benefit to SARS-CoV-2- infected patients when given as post-exposure prophylaxis or early after the onset of clinical symptoms but are of limited or no clinical use during later stages of COVID-19 disease (9, 10).

Antibodies capable of neutralizing SARS-CoV-2 *in vitro* or *in vivo* are directed against the S protein (2, 4, 11–13). Each protomer within the S protein trimer comprises an S1 subunit, which facilitates binding to the cellular receptor ACE2 via a receptor-binding domain (RBD), and an S2 subunit, which drives fusion of the viral envelope with a target cell membrane and harbors a fusion peptide and transmembrane domain. The RBDs are mobile and may bind ACE2 only when in an “up” conformation, compared with the “down” RBD conformation of the prefusion S trimer. Structural and functional analyses have facilitated the identification and classification of neutralization epitopes on the SARS-CoV-2 S RBD (1, 14–17) with the widely used system proposed by Barnes et al. relying exclusively on structural data. According to Barnes and colleagues, class 1 antibodies are frequently derived from VH3-53 related germlines and share a very similar angle of approach to bind to a group of epitopes heavily overlapping with the ACE2 binding site. Class 2 antibodies recognize adjacent epitopes also overlapping with the ACE2 binding site but are more heterogeneous concerning germline usage and angle of approach. The epitopes of class 3 antibodies do not overlap with the ACE2 binding site and usually include the N-glycosylation site at position 343. Finally, class 4 antibodies bind to epitopes located on the inner face of the RBD. Although these epitopes do not significantly overlap with the ACE2 binding site, class 4 antibodies compete with ACE2 by steric hindrance. Although most of the so far reported neutralizing antibodies recognizing the SARS-CoV-2 RBD fall into one of these four classes, a few antibodies recognize additional epitopes that have also been characterized by structural analysis of their binding mode to the RBD (18, 19). A small group of antibodies has been reported to target the more conserved β-strand core of the RBD with an extended CDR2 region (20–26) and neutralize SARS-CoV-2 by destroying the S trimer without interfering significantly with its binding to ACE2.

The rapid evolution of SARS-CoV-2 over the last 3 years has led to the emergence of new SARS-CoV-2 variants, of which several were classified as “variants of concern” (VOC) because of their increased transmission rates (Alpha, Delta, and Omicron), decreased vaccine protection (Beta, Gamma, and Omicron), or increased hospitalization rates (Gamma and Delta). As a result, several of the human nAbs approved for clinical use early on during the SARS-CoV-2 pandemic have been shown to lose their potency when confronted with particular SARS-CoV-2 VOCs, and key mutations conferring neutralization resistance have emerged [reviewed in (10)]. Thus, bamlanivimab/LY-CoV555 is susceptible to the E484K mutation (found in VOCs Beta, Gamma, and sublineages of Alpha), whereas etesivimab/LY-CoV016 is susceptible to K417N and E484K (both found in VOC Beta). Likewise, casirivimab/REGN10933 is susceptible to K417E/N and E484K as well as Y453F (found in SARS-CoV-2 isolates from mink farms), and the efficacy of imdevimab/REGN10987 is compromised by ∆242/244 (some sublineages of Beta), N439K (VOI B.1.258), and others (10). Such resistance-conferring mutations have affected antibodies of all four structural classes, which comprise most of the currently known SARS-CoV-2 neutralizing antibodies. Even the broadly reactive and widely used sotrovimab is inactive against many omicron sublineages (27); see (28) for a comprehensive overview of therapeutic nAb activity on SARS-CoV-2 variants. On the other hand, antibodies such as R1-32, FC08, Ab08, CR3022, EY6A, FD20, and 35B5 that target the more conserved β-strand core are thought to be less sensitive to escape mutants (20–26). However, several members of this group have been found to be susceptible to the substitution of L452 by an arginine (L452R), glutamine (L452Q), or tryptophan (L452W) residue, as found in the VOC Delta, VOI Lambda, and several of the later Omicron variants, such as BA.2.86, XBB.1.5, and DV.7.1 (29) and therefore have a reduced capacity to neutralize these SARS-CoV-2 variants.

Here, we report a new member of this group of RBD core-targeting neutralizing antibodies (pT1616) with very broad reactivity. It retains neutralizing activity against the majority of VOCs and VOIs, including the Omicron subvariants BA.1, BA.2, BA.2.75.2, XBB.1.5, and EG.5.1, and to a limited extent also the VOC Delta and the Omicron variants BA.4/5, BA.4.6, and BQ.1.1. The crystal structure of the SARS-CoV-2 RBD in complex with a pT1616 Fab fragment reveals details of the interactions with the conserved epitope described above. Finally, pT1616 protects Syrian hamsters from SARS-CoV-2-induced pathology *in vivo*. The broad reactivity of this antibody provides insights into the recognition of this particular conserved epitope and sheds light on the neutralizing determinants targeted by human-neutralizing antibodies.

## RESULTS

### Selection of a neutralizing human monoclonal antibody with broad activity against SARS-CoV-2 variants

**Fig 1:**
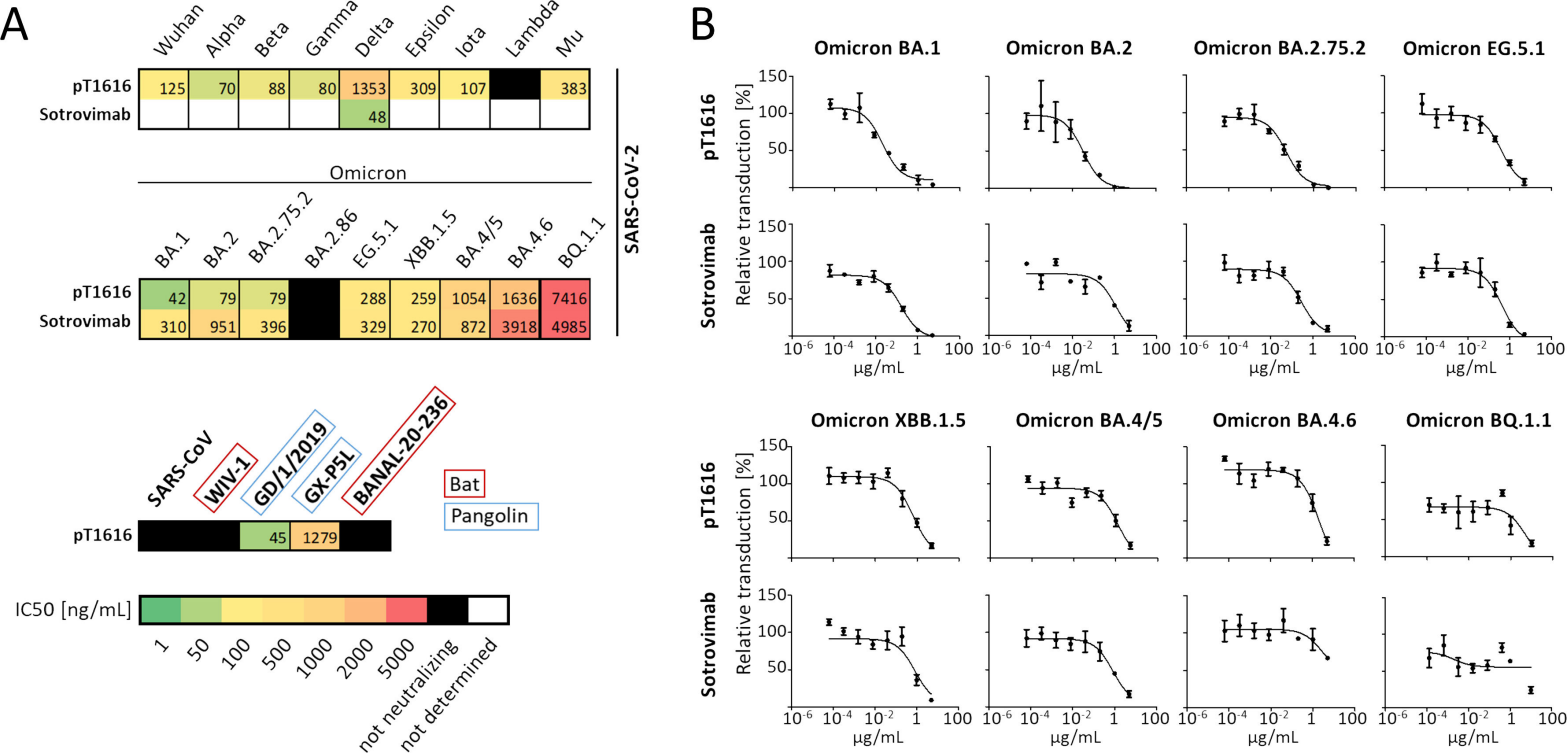
Neutralization of VSV pseudotyped with S proteins from SARS‐CoV‐2 wildtype, SARS-CoV-2 variants, or related sarbecoviruses by IgGs pT1616 and Sotrovimab. (**A**) Heat maps showing the geometric mean of the IC50 calculated from three independent experiments as described in Materials and Methods. (**B**) Neutralization graphs for the Omicron variants, show a representative result of one of three biological replicate experiments used for the IC50 calculation shown in panel A.

We screened sera from 474 patients, who were suffering or had recovered from a clinically manifest SARS-CoV-2 infection, for neutralizing antibodies to SARS-CoV-2 using a previously described VSV pseudotype-based neutralization assay employing a carboxyterminally truncated S protein of the original SARS-CoV-2 Wuhan strain (30). To identify donors whose sera contained antibodies capable of also neutralizing related betacoronaviruses, we further tested 110 of the most potent SARS-CoV-2 neutralizing sera for their ability to also neutralize VSV pseudotyped with the S proteins of the highly pathogenic human betacoronaviruses SARS-CoV, as well as the SARS-CoV-related bat sarbecovirus WIV-1. In this manner, we identified three individuals whose sera reduced SARS-CoV-2 infection by more than 50% at a dilution of 1:1,600 and SARS-CoV and WIV-1 infection to a similar extent at a dilution of 1:25 or higher. We collected peripheral blood mononuclear cells from these individuals and isolated single SARS-CoV-2-reactive memory B cells by a combination of magnetic-activated cell sorting (MACS) and flow cytometry (see Materials and Methods). To enrich mAbs specifically targeting the SARS-CoV-2 S protein, we labeled B cells with a recombinant S protein extracellular domain fused to mNeonGreen. Single-cell sequencing of the selected B cells from all three patients yielded 7,922 productive IgG heavy chains. We cloned 352 heavy- and light-chain paired sequences in the form of single-chain variable fragments (scFv), produced them in *Drosophila melanogaster* S2 cells (31), and tested them for their ability to neutralize VSV pseudotyped with S protein of the SARS-CoV-2 Wuhan strain. We selected 43 neutralizing scFvs and tested them in pseudotype neutralization assays against SARS-CoV-2 VOCs as well as selected VOIs and other variants that had emerged until summer 2021 (Alpha, Beta, Gamma, Delta, Epsilon, Iota, and B.1.258). A panel of 19 nAbs was then expressed as intact IgG1 molecules and tested in pseudotype assays for their neutralizing activity against SARS-CoV-2 Wuhan and the variants Alpha, Beta, Gamma, Delta, Epsilon, and Iota. The best six neutralizing antibodies were further tested against variants Lambda, Mu, B.1.258, and, alongside the clinically approved antibody sotrovimab, the Omicron variants BA.1, BA.2, BA.4/5, BQ.1.1, XBB.1.5, EG.5.1, and BA.2.86. Only one of these six antibodies (pT1616) retained neutralizing activity against Omicron BA.1, BA.2, XBB.1.5, and EG.5.1 (Fig. 1A), and to a limited extent also against BA.4/5 and BQ.1.1. To obtain a complete neutralization profile, this mAb was subsequently also tested against the Omicron variants BA.2.75.2 and BA.4.6. Pseudotype assays for neutralization of several bat and pangolin coronavirus S proteins revealed that pT1616 neutralized the SARS-CoV-2-related pangolin viruses GD/1/2019 and GX-5PL, albeit to a different degree (Fig. 1A), but did not inhibit SARS-CoV and the related bat virus WIV-1. Also, the bat sarbecovirus BANAL-20–236, which is currently the closest relative of SARS-CoV-2 and capable of infecting human cells (32), was not neutralized (Fig. 1A).

### Binding of pT1616 to betacoronavirus S proteins

**Fig 2:**
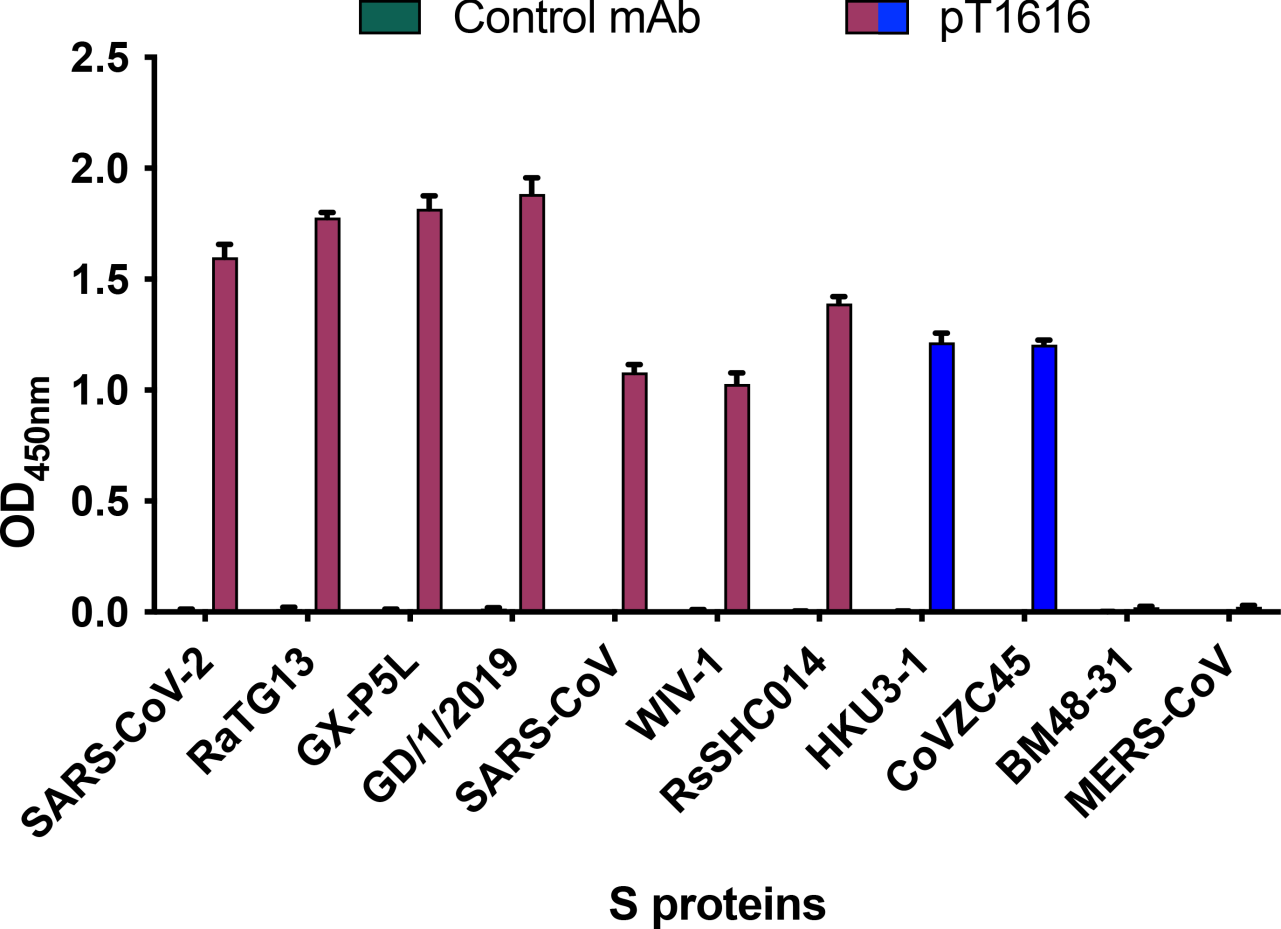
Binding of bnAb pT1616 against selected beta-coronavirus S proteins. Binding of pT1616 to recombinant S proteins from 10 representatives of the sarbecovirus subgenus and one from the merbecovirus subgenus was measured by ELISA. S proteins binding to the angiotensin-converting enzyme 2 (ACE2) are colored in red, and S proteins binding to other receptors in blue. The graph depicts the mean values of four biological replicates (*N* = 4), error bars indicate the standard deviation.

We tested the ability of pT1616 to bind to recombinant S proteins from the merbecovirus and sarbecovirus lineages of the beta coronavirus genus in an ELISA assay. As a representative of the former group, we chose MERS-CoV, and the latter group included S proteins from Asian (CoVZC45, HKU3-1, RsSHC014, and RaTG13) or European (BM48-31) bat sarbecoviruses, whose S proteins do not readily mediate entry into Vero cells and can therefore not be tested in a pseudotype neutralization assay (33–37). pT1616 did not bind to MERS-CoV S protein from the merbecovirus lineage and the sarbecovirus BM48-31 S protein but bound to S proteins from other members of the sarbecovirus lineage, including S proteins that use (RaTG13, RsSHC014, and WIV-1) or do not use (CoVZC45 and HKU3-1) ACE-2 as a receptor (Fig. 2). Determination of kinetic binding parameters of the nAb-S protein interaction using surface plasmon resonance (SPR) with either trimeric SARS-CoV-2 S protein or recombinant RBD revealed dissociation constants *K_D_* in the picomolar range (Fig. S1; Table S3). As expected, pT1616 showed a more than 3-fold lower dissociation constant *K_D_* for binding to the S trimer when compared with the monomeric RBD.

### Structural characterization of SARS-CoV-2 bnAb pT1616

**Fig 3:**
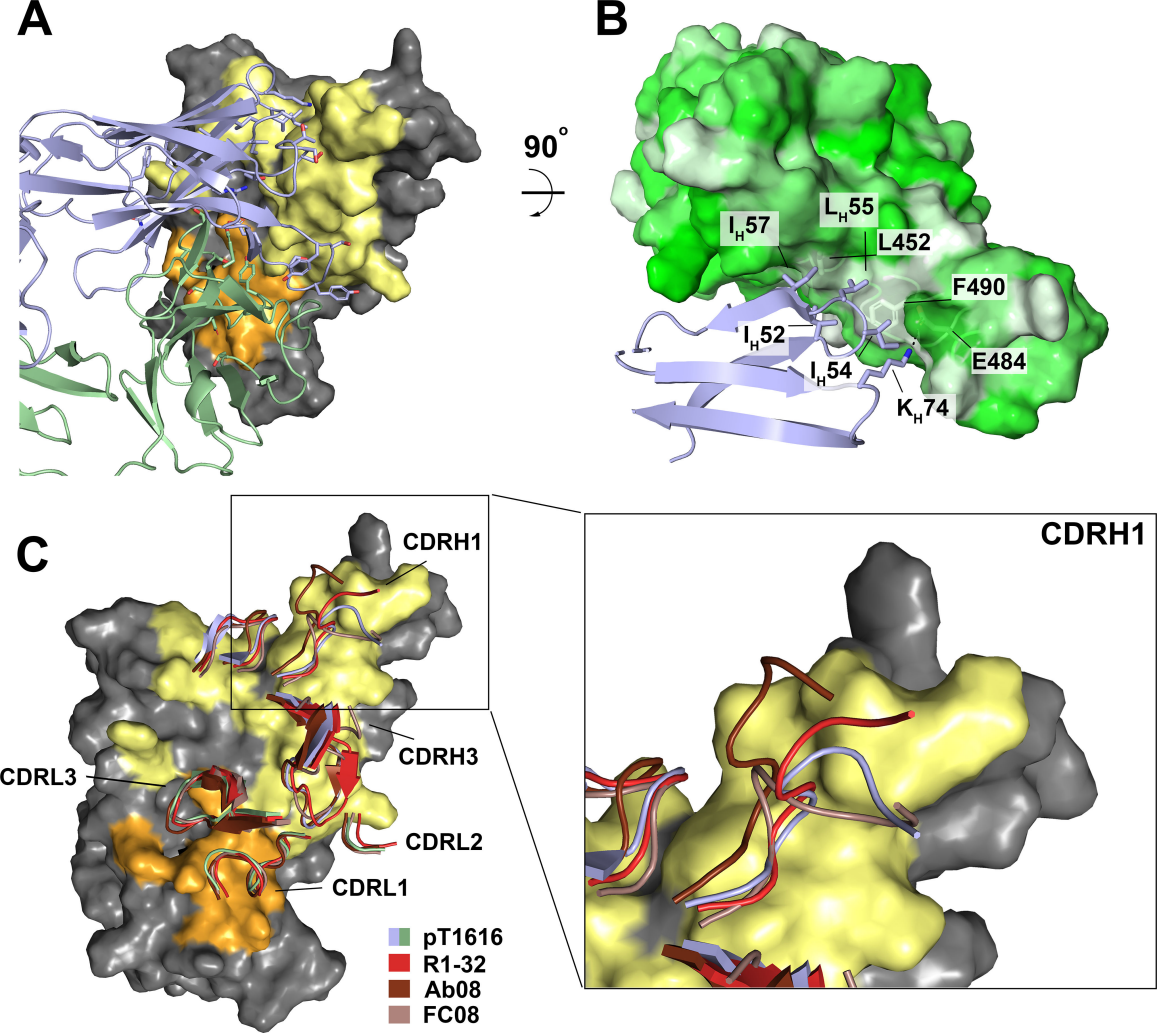
Crystal structure of bnAb pT1616 in complex with SARS-CoV-2 RBD. (**A**) Surface view of SARS-CoV-2 RBD in complex with bnAb pT1616 shown as cartoon colored in light blue (HC) and dark green (LC). Colored regions on the RBD represent residues that are closer than 3.5 Å from pT1616 heavy (yellow) and light chain (orange), respectively. (**B**) The molecular surface of the RBD is colored according to a normalized hydrophobicity scale from white (hydrophobic) to green (hydrophilic). Residues of the pT1616 CDRH2 that are part of the hydrophobic patch and contribute to the interaction with the RBD are shown as sticks. The surface of the two hydrophobic residues within the RBD (L452 and F490) that allow neutralization escape upon mutation is transparent, and the salt bridge between K_H_74 and E484 is indicated as a black dashed line. (**C**) Analysis of the CDR conformations of antibodies Ab08 (PDB 7WQV), FC08 (PDB 7D × 4), R1-32 (PDB 8HC5), and pT1616 (this paper) reveals almost identical CDR backbones for all CDRs except for the CDRH1 (inset).

To better understand the interaction of our strongly neutralizing antibody with the SARS-CoV-2 S protein at the atomic level, we determined the X-ray structure of a pT1616 Fab fragment in complex with a recombinant SARS-CoV-2 RBD to a resolution of 3.11 Å. Details on data collection and structure quality statistics are provided in Table S4. The epitope targeted by pT1616 is located away from the receptor-binding motif (RBM) with a buried surface area (BSA) of 1198 Å^2^ divided in a heavy chain (HC) contribution of 817 Å^2^ (68.2%) and a light chain (LC) contribution of 381 Å^2^ (31.8%; Fig. 3A). The interaction is dominated by contacts of the RBD with the CDRH1, CDRH2, and CDRH3 together with residues within CDRL1 and CDRL3. It can be best categorized as “group E2.1 and C” antibody (17).

Superposition of the pT1616-complex structure onto the SARS-CoV-2 S protein in its stabilized prefusion conformation reveals clashes between the variable region of the LC and the N-terminal domain (NTD) of the neighboring S protein protomer. These clashes are minor in the RBD “up” conformation and are more pronounced in the RBD “down” conformation (Fig. S2). The mode of binding of pT1616 resembles the one observed for the human nAbs Ab08, FC08, and R1-32 (Fig. 3C), which were reported to target a cryptic epitope distant from the RBM (20, 22, 25, 38). These antibodies disrupt S protein trimers, explaining their ability to neutralize SARS-CoV-2 without interfering with the RBD-ACE2 interaction. In view of the similar binding mode antibody, pT1616 will likely act in a similar manner.

Ab08, FC08, R1-32, and pT1616 are all VH_1_-69 antibodies and also encode light chains derived from the same germline gene (VL_1_-40), a combination that is infrequently observed in Abs neutralizing SARS-CoV-2 [five nAbs of 409 structurally characterized nAbs reported by February 2024 (39)]. The selectivity for the VH_1_-69 germline gene can likely be attributed to a hydrophobic patch within CDRH2 that dominates the interaction in this interface region and is not encoded in other germline genes. This hydrophobic patch is key for VH_1_-69 antibodies potently neutralizing other pathogenic viruses like hepatitis C virus (HCV), influenza virus (40), and human immunodeficiency virus (HIV) (41–43). The pT1616 CDRH2 is particularly hydrophobic, with residues I_H_52, I_H_54, L_H_55, and I_H_57 contributing almost a quarter of the total BSA (~247 Å^2^; Fig. 3B). This hydrophobic patch interacts with RBD residues L452, F490, and L492, similar to the previously reported S protein-destructing antibodies Ab08, R1-32, and FC08.

**Fig 4:**
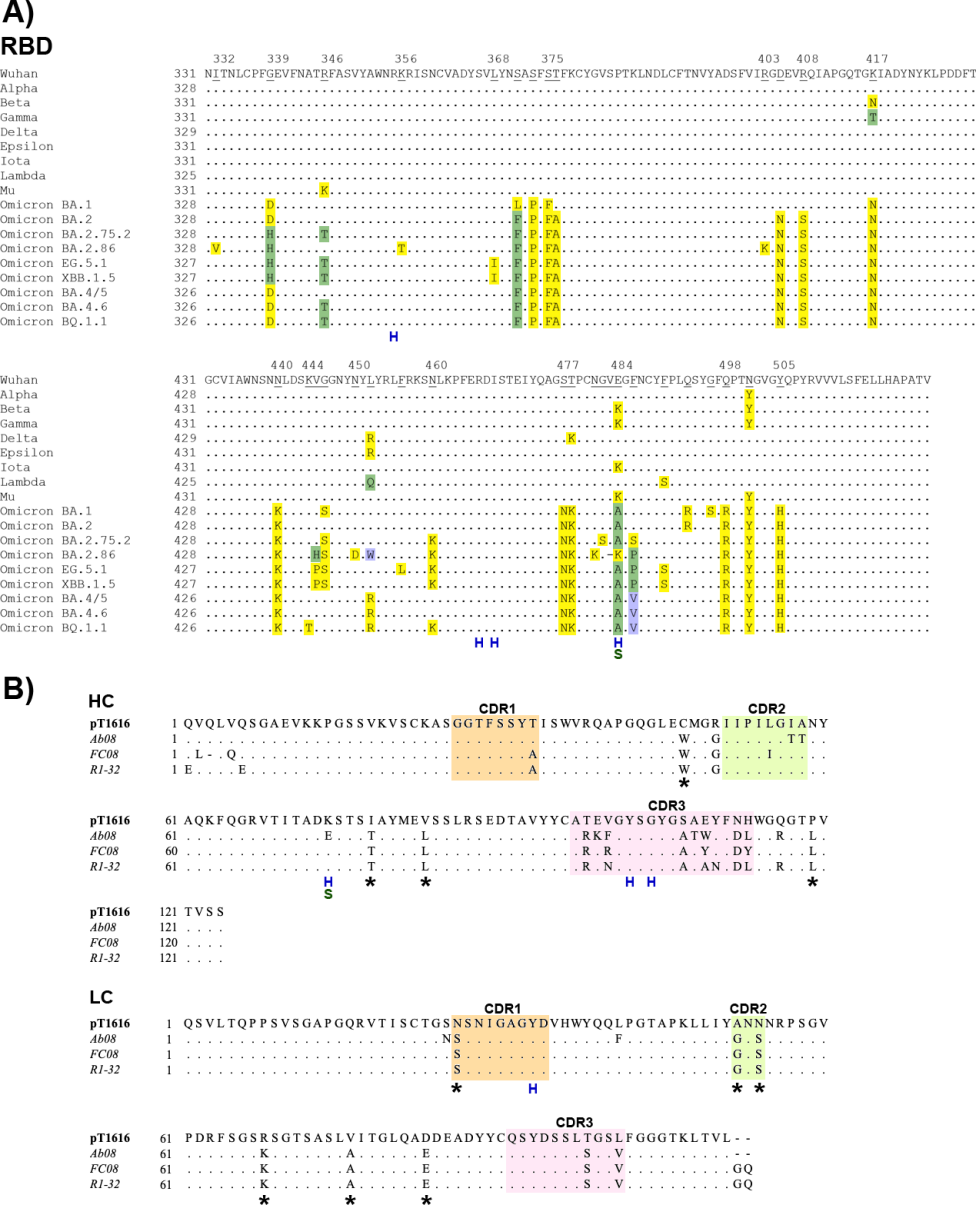
Amino acid alignment of (**A**) S protein RBD sequences of the variants examined in this study and (**B**) the human IGVH1-69 antibodies recognizing the pT1616 epitope. Dots represent conserved residues, residues for which mutations have been reported in at least one of the studied variants are underlined in the top row. Reported amino acid exchanges within the RBD are shaded, with different colors (yellow, light green, and light blue, respectively) representing distinct mutations at individual positions. Hydrogen bonds and salt bridges observed in the two independent copies of the pT1616-complex as detected by the PISA server (46) are indicated in blue (hydrogen bonds) and green (salt bridges). CDR1, CDR2, and CDR3 are colored in sand, light green, and light pink, respectively, in heavy and light chains. Somatic mutations outside the antibody CDR3 regions are indicated by an asterisk.

Mutations of residue L452 have frequently occurred in SARS-CoV-2 variants including Delta (L452R), Epsilon (L452R), Lambda (L452Q), and later on in Omicron variant BA.4 and BA.5 (L452R) (44, 45) and their descendant BQ.1 (L452R) (44) (Fig. 4). For reported antibodies targeting the pT1616 epitope, L452 is of critical importance for efficient neutralization as their activity is markedly compromised by substitutions at this position. A single L452R mutation led to >20-fold reduced neutralization by Ab08 (22) and a ~ 23-fold and ~70-fold reduced binding affinity of R1-32 and FC08, respectively (20). In contrast, we show that a pseudotyped virus carrying an S protein with the L452R substitution (corresponding to the Epsilon variant) is neutralized by pT1616 almost as efficiently as a pseudotype carrying the Wuhan S protein (Fig. 1). An alignment of RBD sequences used in this study (Fig. 4) together with our neutralization results shown in Fig. 1 suggest that a more drastic disruption of the interaction between the hydrophobic patch in CDRH2 and the RBD, as observed in Lambda (L452Q & F490S via the accumulation of a second substitution in the interface) and BA.2.86 (L452W via the presence of a very large, bulky sidechain), was needed to facilitate escape from pT1616 neutralization (Fig. 1).

Antibodies targeting the pT1616 epitope do not differ drastically in their CDRH3 sequences (Fig. 4B), and light chain sequences are almost identical. To understand the observed functional differences between individual antibodies in tolerating interface substitutions within their respective epitopes, we compared the pT1616-RBD interface in more detail with the one of Ab08-RBD, which has been structurally characterized up to a resolution of 2.8 Å. The two antibodies differ in a prominent residue (K_H_74 in pT1616 vs. E_H_74 in Ab08) within the heavy chain framework region that appears to form a salt bridge with E484 in the pT1616-complex (Fig. 3B). In addition, the overall stability of the complex caused by hydrophobic interactions is predicted to be higher for the pT1616-complex than for the Ab08-complex as judged by the higher predicted solvation free energy gain upon complex formation [average Δ_i_G for the independent complexes in the asymmetric unit; −5.5 kcal/mol vs. −4.5 kcal/mol for pT1616 and Ab08, respectively; PISA server (46)]. Eventually, comparison of the CDR backbone orientation revealed that primarily the CDRH1 backbone differs not only between pT1616 and Ab08 but also between pT1616 and the other two trimer-disrupting antibodies (Fig. 3C), and the interaction between CDRH1 and the RBD appears more extensive in the pT1616 complex than in the Ab08 complex, both in terms of buried surface area and the number of hydrogen bonds formed in the interface.

In summary, the higher escape barrier of pT1616 is likely caused by several factors including the salt bridge formed by K_H_74. This combination of interface features in pT1616 likely results in a higher affinity interaction and a higher tolerance for escape mutations, leading to a broader neutralization profile of VOCs and VOIs than Ab08 despite a similar binding mode.

### Broadly nAb pT1616 protects against SARS-CoV-2 challenge in a hamster model

**Fig 5:**
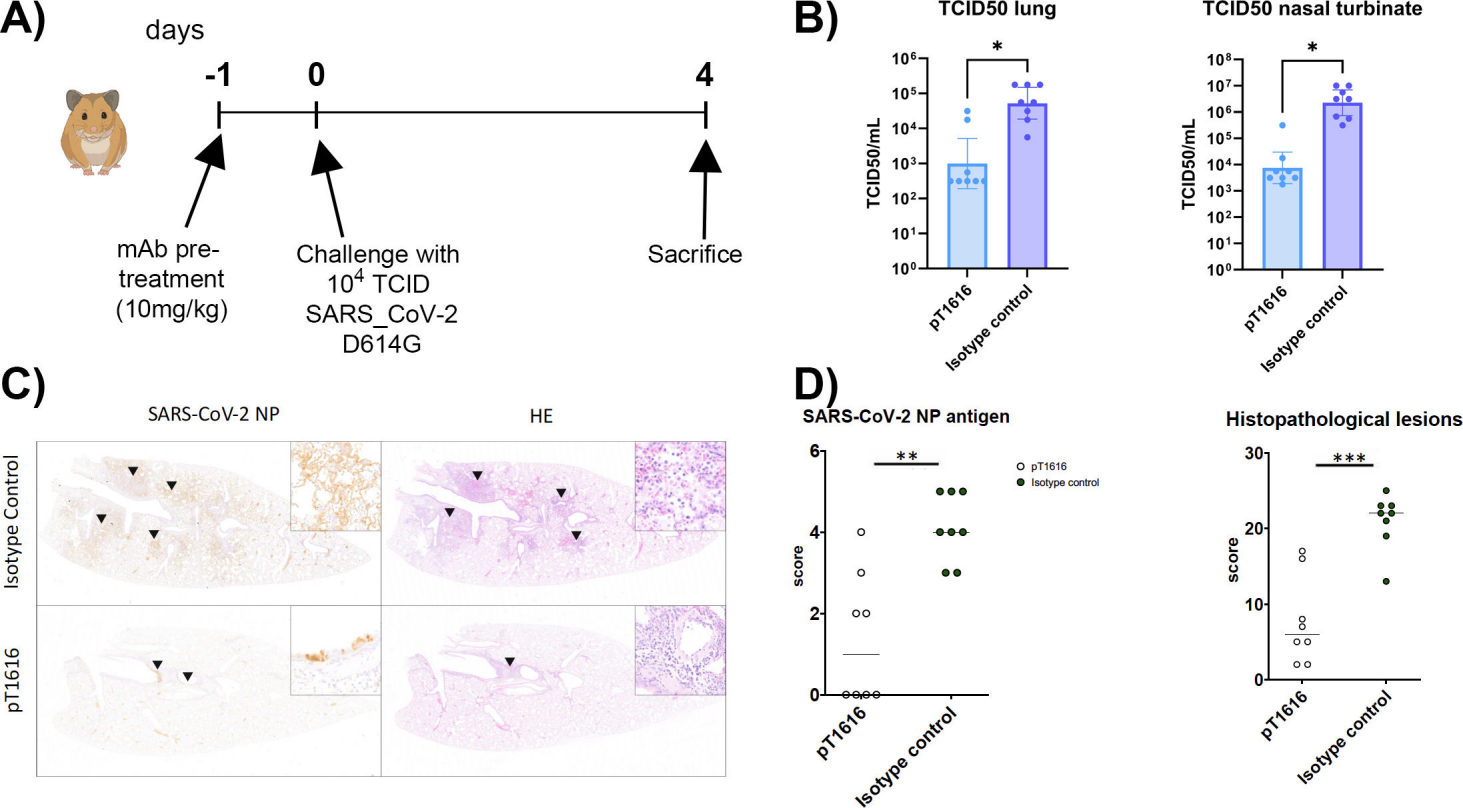
Protection of Syrian hamsters against SARS-CoV-2 infection and disease by nAb pT1616. (**A**) Study design to test the protective effect of pT1616 on SARS-CoV-2 infection. (**B**) Viral load, measured as TCID50, in the lung (left) and nasal turbinates (right) of Syrian hamsters pretreated with 10 mg/kg pT1616 antibody or an isotype control antibody before intranasal challenge with 10^4^ TCID50 of a SARS-CoV-2 b.1/D614G isolate 24 h later. Shown is the geometric mean with 95% confidence intervals. Significant differences between control and treated groups are labeled with an asterisk (*P < 0.05; unpaired two-tailed *t*-test). (**C**) Immunohistochemistry for SARS-CoV-2 nucleoprotein (NP) (left) and hematoxylin/eosin (HE) staining (right) of lung tissue sections from animals treated with an isotype control or the indicated nAbs. Arrowheads indicate SARS-CoV-2 NP immunolabelled cells (left panels) or histopathological lesions characterized by epithelial degeneration and necrosis with immune cell infiltration. (**D**) Semi-quantitative analysis of SARS-CoV-2 immunolabelled cells (left), and histopathological score to assess lesion severity (right), in lung sections of SARS-CoV-2 infected hamsters from the different treatment groups. Significant differences between control and treated groups are labeled with asterisks (*P < 0.05, **P < 0.05, ***P < 0.001, Kruskal-Wallis test).

We next tested whether our broadly nAb pT1616 would protect against a SARS-CoV-2 challenge in an animal model for moderate-to-severe COVID-19 (47). The hamster model is permissive to all SARS-CoV-2 variants, including earlier (Wuhan) and later (Omicron) variants (47, 48). We pretreated hamsters by i.p. injection of 10 mg/kg purified antibody pT1616 or an isotype control and challenged them with 10^4^ TCID50 of a SARS-CoV-2 B.1/D614G isolate 24 h later (Fig. 5A), as described previously (49). The animals were sacrificed on day 4. Viral load in lung homogenates and nasal turbinates, measured as TCID50, was reduced by about 1–2 logs in animals pretreated with neutralizing antibodies compared with isotype control-treated animals (Fig. 5B). To confirm these data and assess viral antigen distribution within the lung, we immunolabeled lung sections with an antibody against the SARS-CoV-2 nucleoprotein (NP). Isotype control-treated animals showed multifocal areas with numerous immunolabeled pneumocytes (types I and II) and bronchial epithelial cells (Fig. 5C; top left), whereas animals treated with pT1616 showed a reduced number of immunolabeled cells in the alveoli, staining restricted to bronchial epithelium or a complete lack of signal throughout the entire investigated lung section (Fig. 5C; bottom left). Semi-quantitative evaluation of SARS-CoV-2 NP immunolabeled sections (49) revealed significantly lower amounts of viral antigen in animals pretreated with antibody pT1616 compared with isotype control-treated animals (Fig. 5D; left).

On HE-stained lung sections, animals treated with the isotype control antibody showed multifocal, large areas of histopathological changes including infiltration of immune cells that were affecting alveoli and bronchi (Fig. 5C; top right). Lung sections of animals treated with pT1616 showed only focal areas with bronchitis (Fig. 5C; bottom right) and a notable sparing of alveoli. When quantified using a semi-quantitative score (37), these histopathological lesions were significantly reduced in animals pretreated with antibody pT1616, compared with isotype-treated controls (Fig. 5D; right).

## DISCUSSION

It is now well established that neutralizing antibodies to SARS-CoV-2 can protect against infection as well as COVID-19 disease when given as post-exposure prophylaxis or early after the onset of clinical symptoms (10). However, during the first 3 years of the SARS-CoV-2 pandemic, successive SARS-CoV-2 variants have emerged that show resistance to the first generation of clinically approved antibodies or antibody combinations. Even antibodies such as sotrovimab, which retained potent neutralizing properties against SARS-CoV-2 Delta and Omicron BA.1, are now less effective against SARS-CoV-2 Omicron BA.2, BA.4/5, and XBB sublineages (27, 50, 51), and resistance against sotrovimab can develop in immunocompromised patients (52). If nAbs are to remain an effective instrument for treating or preventing COVID-19 disease, we need to better understand virus evolution and determinants of broad and potent neutralization, allowing for the identification of broadly nAbs that retain their potency against previous, current, and future SARS-CoV-2 variants. Although recently antibodies with very broad neutralization profiles have been reported (53, 54) that target the fusion peptide, the neutralizing potency and therapeutic potential of these antibodies is lower than that of efficient RBD-targeting antibodies. Furthermore, in view of the emergence of three highly pathogenic human betacoronaviruses with pandemic potential over the last 2 decades and the sporadic transmission of additional animal coronaviruses to humans (55, 56), the likelihood of another zoonotic outbreak of an as-yet-unknown coronavirus is high. Identifying and stocking human bnAbs capable of inhibiting related betacoronaviruses could therefore represent an important contribution toward “epidemic preparedness.”

To understand the determinants to neutralize SARS-CoV-2 variants on the one hand and related sarbecoviruses on the other hand, we characterized human nAbs isolated from “elite neutralizers.” We identified one antibody (pT1616) that retained its ability to neutralize almost all successive SARS-CoV-2 variants in a pseudotype neutralization assay, including the Omicron subvariants BA.1, BA.2, BA.2.75.2, and EG.5.1 and to a lesser extent also BA.4/5, BA.4.6. and XBB.1.5. This antibody recognizes a cryptic epitope that does not fall within the widely used Barnes classification and is located on the lateral face of the RBD, away from the RBM. The same epitope has previously been shown to be recognized by three nAbs Ab08, FC08, and R1-32 (20, 22, 25) that do not interfere with the RBD-ACE2 interaction; instead, they have been shown to destabilize the S protein trimer. Like pT1616, all three antibodies are VH_1_-69 antibodies, suggesting a strict requirement for this germline to bind to this epitope that can be explained by the hydrophobic CDRH2 unique to this germline gene binding to a hydrophobic patch on the RBD surface encompassing three hydrophobic residues (L452, F490, and L492). While the previously reported representatives of this class of SARS-CoV-2 nAbs are highly sensitive to mutations of L452, such as L452R, antibody pT1616 only loses its potency either due to an amino acid exchange into a large bulky residue like L452W (BA.2.86) or an additive effect on the hydrophobic interaction with the CDRH2 by a second amino acid exchange at position F490S (Lambda; Fig. 1 and 4). Our findings underline that although recognizing its cryptic epitope in a similar manner as the previously described antibodies Ab08, FC08, and R1-32, minor differences in the pT1616 footprint translate into a significantly broader neutralization potential in particular with a higher resistance to the L452R mutation, highlighting the importance of such a careful analysis of the structure-function relationship for individual antibodies.

## MATERIALS AND METHODS

### Cell culture

All cell lines were cultivated at 37°C in a humidified atmosphere with 5% CO_2_. HEK293T, Vero76, and BHK-21(G43) were grown in Dulbecco’s modified Eagle medium (DMEM, Gibco 41966–029) with 10% fetal bovine serum (FBS, Sigma F7524). Vero76 cells were supplemented with 100 U/mL of penicillin and 0.1 mg/mL of streptomycin (Cytogen, 06–07100). BHK-21(G43) cells were supplemented every fourth passage with 100 µg/mL zeocin and 50 µg/mL hygromycin. Cells were subcultured 1:10 twice (HEK293T, BHK-21(G43)) or thrice (Vero76) a week. For subculture and seeding of cells, the cells were first washed with phosphate buffered saline (PBS) before detaching them with trypsin/EDTA (Cytogen, 10–023).

### Pseudotype neutralization assays

Expression vectors for the S proteins of SARS-CoV, SARS-CoV-2 Wuhan, and the SARS-CoV-2 variants Gamma, Omicron BA.1 (B1.1.529), BA.4/5, BQ.1.1, XBB.1.5, BA.2.86, EG.5.1, BA.2.75.2, BA.4.6, and VSV-G were described previously (30, 57–68). The codon-optimized DNA coding for other coronavirus S proteins used in this study (see Table S1) was ordered from GeneArt (ThermoFisher) and subcloned in the mammalian expression vector pCG1 (kindly provided by Roberto Cattaneo, Mayo Clinic College of Medicine, Rochester, MN, USA) using *Bam*HI and *Xba*I restriction sites. To enhance infectivity of the VSV pseudotypes, the nucleotides coding for the last 18 amino acids of the intracellular C-terminal domain of the S proteins were deleted from the expression construct (69). The protocol and components for the VSV pseudotype neutralization assay have been previously described (70). To produce 2 mL of supernatant containing VSV pseudoparticles, 4 × 10^5^ HEK293T cells were plated per well in a 6-well dish in a 2 mL culture medium containing 25 mM HEPES. The next day, the cells were transfected via calcium phosphate transfection with 8 µg of the desired surface glycoprotein expression plasmid. Eighteen hours after transfection, the transfected cells were transduced by VSV*ΔG-fLuc(VSV-G), a VSV vector in which the VSV-G open reading frame has been replaced by an expression cassette for eGFP and firefly luciferase and which was produced and complemented with VSV-G in BHK-21(G43) cells. After a 2-h incubation period with the vector at 37°C, 5% CO_2_, the cells were washed twice with PBS. To neutralize residual virus left from the transduction, 2 mL culture medium with 25 mM HEPES, and anti-VSV-G antibody (I1, mouse hybridoma supernatant from CRL-2700; ATCC) was added per well, except for the wells where the control virus containing the VSV-G protein was produced. Twenty-four hours after transduction the pseudotype containing supernatant was cleared of cells by centrifugation and subsequently stored at 4°C until used in the neutralization experiments.

For use in the neutralization assays, 1 × 10^4^ Vero76 cells per well were plated in 96-well plates the day before the assay. Supernatant containing pseudotype particles was mixed with human heat-inactivated (56°C, 30 min) serum, scFv, or IgG dilutions and incubated at 37°C for 30 min, before being added to the cells in triplicate wells. Twenty hours later, the cells were lysed (1× Lysis-juice, PJK, 102517) for 30 min, the lysate transferred to opaque white plates, and luciferase activity was measured using a firefly luciferase substrate (Beetle-juice, PJK, 102511) and the Orion II (Berthold) or GloMax (Promega) luminometers. The relative transduction efficiency was calculated after deducting background signal from non-infected wells, by setting the signal from pseudotype virus- infected, but not neutralized, wells to 100% and calculating the percentage of the luciferase values for the wells that received the neutralization mixture. The data were plotted with GraphPad Prism5, and the NT50 and NT90 thresholds were marked.

To determine IC50 values for individual neutralizing antibodies, we used pseudotype virus preparations with TCID50 values in the range of 10^4^/mL, with the exception of the BANAL-20–236 S protein pseudotype virus, which was less infective and could only be used at a maximal TCID50 of 10^3^ /mL. TCID50 values were determined on Vero76 cells plated in 96-well plates on the day of the neutralization assay. Individual neutralizing IgGs were serially diluted from a starting concentration of 5,000 ng/mL in eight 5-fold dilution steps to a concentration of 0.064 ng/mL. Following the deduction of background Firefly luciferase values, IC50 values were calculated using the GraphPad Prism5s nonlinear regression feature with the log(inhibitor) versus response (three parameters) equation and a bottom constrained to zero to account for the background deduction. The heatmaps shown in Fig. 1A are based on the geometric mean of three independent experiments.

### B-cell sorting and single-cell sequencing

Frozen PBMCs from three donors were thawed on ice and resuspended in 20–30 mL degassed MACS buffer (PBS, pH 7.2, 2 mM EDTA, 0.5% (wt/vol) BSA) after written consent by the participants. Cells were centrifuged at 300 × *g* and 4°C for 20 min, resuspended in 500 µL MACS buffer, and live cells were counted with the Countess II cell counter (40: 4.6 × 10^7^ cells, 54: 8.43 × 10^7^ cells, and 17: 3.51 × 10^7^ cell). Cells were pelleted (350 × *g*, 4°C, 10 min) and prepared for magnetic cell separation using human CD19 microbeads (Miltenyi Biotec) and LS columns (Miltenyi Biotec) following the manufacturer’s instructions. For FACS, MACS-sorted cells were labeled with 0.2 mg/mL SARS-CoV-2-mNeon-fused S protein (Wuhan), 20 µL APC Mouse anti-Human IgG (BD Bioscience), and 5 µL Alexa Fluor 700 Mouse anti-Human CD20 (BD Biosciences) per 10^6^ cells in 100 µL, in addition to LIVE/DEAD Fixable Near-IR Dead Cell Stain (ThermoFisher Scientific). As a negative control, a mNeon-labeled unrelated viral glycoprotein protein was used to test for unspecific mNeon-binding. During labeling, cells were incubated for 30 min on ice and later washed with MACS buffer before resuspending in 400 µL PBS supplemented with 0.5% (wt/vol) BSA. Cells were then sorted on a BD Bioscience FACSAria III Fusion sorter, and the Chromium Next GEM Single Cell V(D)J Reagent Kits v1.1 was used to process the single-cell solutions. Sequencing of the scBCR libraries was performed on the Illumina NextSeq550 platform using the NextSeq 500 Mid Output Kit v2.5. The flow cytometry data were analyzed using the FCS Express 7 software, whereas the sequencing data were analyzed with the Loupe V(D)J Browser, and productive sequences were re-annotated with IMGT/HighV-Quest.

### Expression and purification of soluble S proteins and SARS-CoV-2 RBD in insect cells

All genes encoding the ectodomains of S proteins from the selected betacoronaviruses (Table S1) and the SARS-CoV-2 RBD (aa 334–527) were cloned into a pMT vector for expression in *Drosophila* S2 cells as described previously (71). Expression constructs were designed to allow expression of the SARS-CoV-2, SARS-CoV, and MERS-CoV S trimers in prefusion conformation as previously described (72–74). However, in addition to a T4 fibritin trimerization motif, a TEV protease cleavage site, a fluorescent protein (mNeon-green), and a double strep-tag were included at the C-terminal end of the ectodomains. All other S proteins were expressed as wildtype proteins. The SARS-CoV-2 RBD carried an enterokinase cleavage site (EK) and a double strep-tag at the C terminus. All genes were inserted downstream of the BiP signal sequence to allow secretion of the proteins into the S2 cell media. A stable S2 cell transfectant was established per construct and the proteins produced as recently described (75). S trimers and SARS-CoV-2 RBD proteins were affinity purified from the S2 cell media using a Strep-Tactin XT 4Flow column (IBA Lifesciences), followed by size exclusion chromatography using a Superose 6 increase and a Superdex 75 increase 10/300 columns (Cytiva), respectively, equilibrated in 2 mM Tris pH 8 and 200 mM NaCl (for S protein) or 20 mM HEPES pH 7.4 and 150 mM NaCl (for the SARS-CoV-2 RBD). Purified proteins were concentrated and stored at −80°C.

### Expression of soluble SARS-CoV-2 S protein trimer in HEK cells

SARS-CoV-2 HexaPro Spike-coding plasmid was a gift from Jason McLellan (Addgene plasmid # 154754). HEK293ExPi cells were transfected using ExpiFectamine 293 transfection reagent (ThermoFisher Scientific) following the manufacturer’s recommendation with minor method modifications. Briefly, a plasmid-DNA cocktail containing the SARS-CoV-2 HexaPro Spike-expressing plasmids, and plasmids encoding cell cycle inhibitors p21 and p27, as well as the large T antigen of the SV40 virus at ratios of 0.69: 0.05: 0.25: 0.01, respectively, was prepared in 5 mL Opti-MEM (Gibco) at a concentration of 1 µg/mL of the final culture volume. The ExpiFectamine 293 transfection reagent was diluted in 5 mL Opti-MEM and allowed to incubate for 5 min at room temperature prior to mixing with the plasmid DNA cocktail. Following a 20-min incubation at room temperature, the transfection mixture was added dropwise to the cells followed by a 16-h incubation before the addition of enhancers. Transfected cells were then incubated for another 4 days after which cells were pelleted, and the soluble trimeric S protein was affinity purified from the supernatant as described for S protein from insect cells.

### Expression and purification of scFv and Fab

Selected single-chain variable fragments (scFv) were assembled by covalently linking the C-terminus of the heavy chain variable regions to the N-terminus of the respective light chain variable regions using a 20 amino-acid flexible linker as described before (31). Codon-optimized synthetic genes of the covalently linked paired scFV or Fab sequences, cloned into a pMT vector were purchased from Twist biosciences. Constructs carried an EK cleavage site and a double strep-tag at the C-terminus of the scFV or at the C-terminus of the Fab heavy chain. Expression of all constructs followed the S2 cell expression protocol described above. The antibodies were affinity purified on Strep-Tactin XT 4Flow column from the S2 cell media followed by SEC using a Superdex 200 increase 10/300 column (Cytiva) equilibrated with PBS. Purified proteins were concentrated and stored at −80°C.

### IgG expression and purification

Paired heavy and light chain variable regions of antibodies were amplified from the respective top neutralizing scFv and cloned into a pcDNA3.1 expression vector under the control of a CMV promoter. All mAbs were expressed as IgG1 in HEK293ExPi cells as described above, and cells were pelleted 5 days post-transfection. IgG was purified by affinity chromatography from the supernatant using a Protein G column (Cytiva), followed by SEC using a Superdex 200 increase 10/300 column (Cytiva) equilibrated with PBS. Purified mAbs were concentrated and stored at 4°C.

### ELISA binding assay

Nunc 96-well ELISA plates (Thermo Scientific) were coated with 100 ng of purified S trimers per well in PBS at 4°C overnight. Plates were washed 3× with 300 µL PBS + 0.05% Tween 20 (PBS-T) and blocked with 100 µL blocking buffer (5% wt/vol skimmed milk in PBS-T) per well for 2 h at room temperature. The plates were washed once with PBS-T, and wells were loaded with 50 µL 2 µg/mL antibody diluted in blocking buffer. After a 30-min incubation at room temperature, the plates were washed 4× with 300 µL PBS-T followed by a 30-min incubation at room temperature with 50 µL per well of horse-radish peroxidase conjugated goat anti-human antibody diluted 1:40,000 in blocking buffer. The plates were then washed 4× with PBS-T and developed by adding 100 µL TMB substrate (BioLegend) per well. The reaction was stopped after 10 min by the addition of 50 µL 1 M H_3_PO_4_ acid and absorbance was measured at 450 nm with 630 nm as reference using the ELx808 absorbance plate reader (BioTek). Data were analyzed using GraphPad Prism (GraphPad).

### Surface plasmon resonance

SPR experiments were performed on a Biacore 3000 (GE Healthcare) at 25°C in 10 mM HEPES (pH 7.4), 150 mM NaCl, 3 mM EDTA, and 0.05% Tween20. The surface of a CM5 sensor chip (Cytiva) was coated with StrepTactin XT (IBA Life Sciences) following the amine-coupling protocol from the Twin-strep-Tag capture kit (IBA Life Sciences). For each analysis cycle (performed in duplicates), the SARS-CoV-2 RBD was injected at a concentration of 3.3 nM for 30 s at a flow rate of 10 µL/min, leading to a reproducible immobilization level of 25 RU. Kinetic data were collected by injecting mAbs at concentrations of 75, 50, 25, 10, and 5 nM for 200 s at a flow rate of 30 µL/min, followed by washing the chip surface with running buffer until a stable baseline was reached. Regeneration was performed after each cycle by injecting 30 µL 3 M guanidine hydrochloride at a flow rate of 30 µL/min. To measure kinetics of the mAbs to the S protein, insect cell-expressed SARS-CoV-2 S protein was injected at a concentration of 100 nM for 30 s at a flow rate of 10 µL/min followed by injection of the mAbs as described for the RBD. Data were analyzed using the BiaEvaluation software with fits to the Langmuir binding equation for a 1:1 interaction model.

### Complex formation, crystallization, and X-ray structure determination

To facilitate crystallization, purification tags were removed from SARS-CoV-2 RBD and Fab preparations using EK according to the manufacturer’s instructions. Uncleaved fusion proteins and free purification tags were removed using a Strep-Tactin XT 4Flow column and subsequent SEC as described above. For complex formation, the pT1616 Fab was incubated with RBD at a molar ratio of 1:1 for 16 h at 4°C, and the resulting complexes were purified using a Superdex 200 increase 10/300 column equilibrated with 10 mM Tris pH 8, 150 mM NaCl. Purified complexes were concentrated and used for crystallization. Crystals of the SARS-CoV-2 RBD in complex with pT1616 Fab were obtained at 20°C by sitting-drop vapor diffusion. Purified antibody complex was mixed with reservoir solution at a 1:1 molecular ratio with a final drop volume of 300 nL. Crystals were obtained using pT1616-RBD at 6.3 mg/mL and 30% PEG 3000, 200 mM NaCl, and 100 mM Tris pH 7.0 as reservoir solution. Crystals were flash-cooled in liquid nitrogen using the reservoir solution supplemented with 30% ethylene glycol as cryoprotectant. Diffraction data were collected remotely at Swiss Light Source beamline PXI. The structure was determined with XDS (76) for processing of data sets and Phaser (77) for molecular replacement using the following modified search models: RBD: 7KGJ, pT1616: 7N4J (light chain) and 7YDY (heavy chain). Several subsequent rounds of model building and refinement were performed using Coot (78) and Phenix (79), with validation using Molprobity (80). See Table S4 for details on data collection, processing, refinement, and validation.

### Hamster challenge experiment

During the experiment, the animals were under veterinary observation, and all efforts were made to minimize distress. Approval for the experiments was given by the German Niedersächsisches Landesamt für Verbraucherschutz und Lebensmittelsicherheit (LAVES file number 20/3493 and 21/375). Syrian hamsters (*Mesocricetus auratus*, 6–10 weeks old, Janvier Labs) were housed under BSL-3 conditions, starting 10 days prior to the experiment. Antibodies were injected intraperitoneally in a volume of 500 µL. The hamsters were challenged intranasally with 10^4^ TCID50 of a SARS-CoV-2 D614G or an Omicron BA.1 isolate. Antibody injection, virus challenge, and euthanasia were performed under isoflurane anesthesia. The animals were monitored for body weight loss and clinical symptoms twice daily until they were humanely euthanized 4 days after infection.

Infectious SARS-CoV-2 virus particles were quantified as previously described (49). In short, lung and nasal turbinate tissues were collected after necropsy and homogenized using a TissueLyser II (Qiagen). Calu-3 cells were infected with 10-fold serial dilutions of the homogenized tissue prepared in MEM + 2% FBS (starting dilution 100-fold and 10-fold for lung and nasal turbinate homogenate, respectively). Plates were further incubated in a humidified atmosphere, at 37°C, 5% CO_2_. Five days after infection, cells were fixed with 4% PFA and stained using an anti-SARS-CoV-2 nucleocapsid antibody (Sinobiological). Virus titers (TCID50/mL) were calculated using the Spearman-Karber method. Left lung lobes from the investigated hamsters were fixed in 10% buffered formalin (Chemie Vertrieb GmbH & Co Hannover KG, Hannover, Germany) and pre-fixed by injections of 10% buffered formalin to ensure an optimal histopathological evaluation (81). Formalin-fixed, paraffin-embedded (FFPE) tissue was used for histology and immunohistochemistry. Histopathological lesions were evaluated on hematoxylin-eosin (HE) stained sections. For the detection of viral antigen in Syrian golden hamsters, immunohistochemistry of SARS-CoV-2 nucleoprotein (NP) was performed using the Dako EnVision + polymer system (Dako Agilent Pathology Solutions) and 3,3´-Diaminobenzidine tetrahydrochloride (Sigma-Aldrich, St. Louis, MO, United States) as previously described (48, 82). Monoclonal mouse primary antibody against SARS-CoV-2 NP (Sino Biological, Peking, China-40143-MM05; dilution 1:16000) was applied overnight at 4°C. Hamster lungs were evaluated on one cross-section (at the level of the entry of the main bronchus) and one longitudinal section (along the main bronchus) of the entire left lung lobe. Two semi-quantitative scoring systems, one for the assessment of histopathological lesions (48, 83) and one for viral antigen distribution (49) in the lung were performed as previously described. In particular, lung slides were evaluated in a blinded fashion and scored by FA and GB. Subsequently, histopathological evaluation and scoring were reviewed and confirmed by board-certified veterinary pathologists (WB, MC).

## Data Availability

The atomic coordinates and structure factors for the crystal structures were depositedin the Protein Data Bank (http://www.pdb.org/) under the accession number 8RRN.

## SUPPLEMENTAL MATERIAL

Supplemental material jvi.01223-24-s0001.pdf Figures S1 and S2; Tables S1 to S4.

ASM does not own the copyrights to Supplemental Material that may be linked to, or accessed through, an article. The authors have granted ASM a non-exclusive, world-wide license to publish the Supplemental Material files. Please contact the corresponding author directly for reuse.
