# Supplementary material for: A human monoclonal antibody neutralizing SARS-CoV-2 Omicron variants containing the L452R mutation
Source: J Virol. 2024 Nov 4;98(12):e01223-24. doi: 10.1128/jvi.01223-24 (PMC11650997; doi:10.1128/jvi.01223-24)
Supplement: Supplemental material — Figures S1 and S2; Tables S1 to S4. [file jvi.01223-24-s0001.pdf]

Supplementary Materials for

A human monoclonal antibody neutralizing SARS-CoV-2 Omicron variants containing the L452R mutation

Saskia C. Stein, Guido Hansen, George Ssebyatika *et al.*

*Corresponding authors. Email: schulz.thomas@mh-hannover.de; thomas.krey@uni-luebeck.de

This PDF file includes:

Figs. S1 to S3
Tables S1 to S4

Supplementary References

Figure S1: Kinetic binding analysis of pT1616 with SARS-CoV-2 RBD and S trimer

A

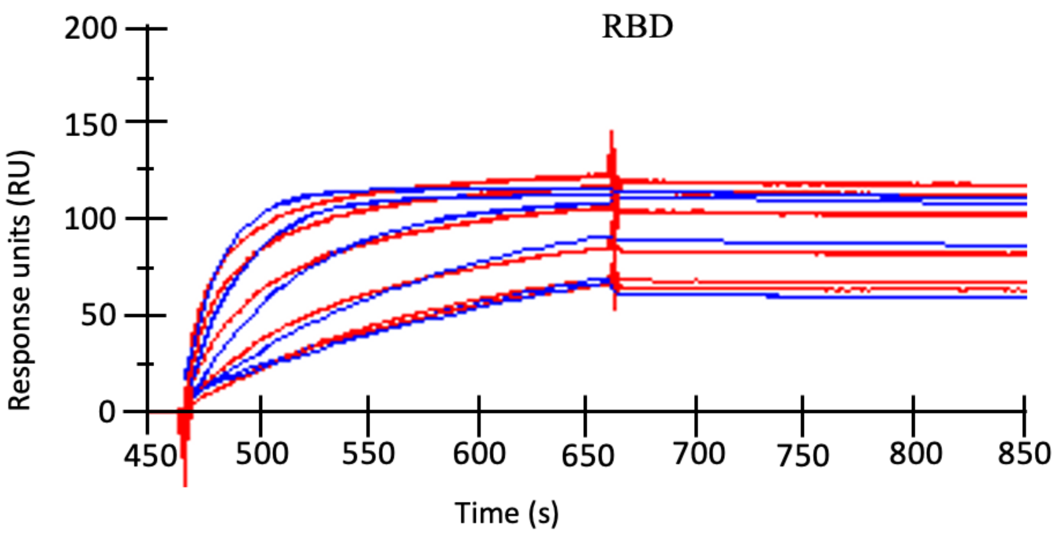

B

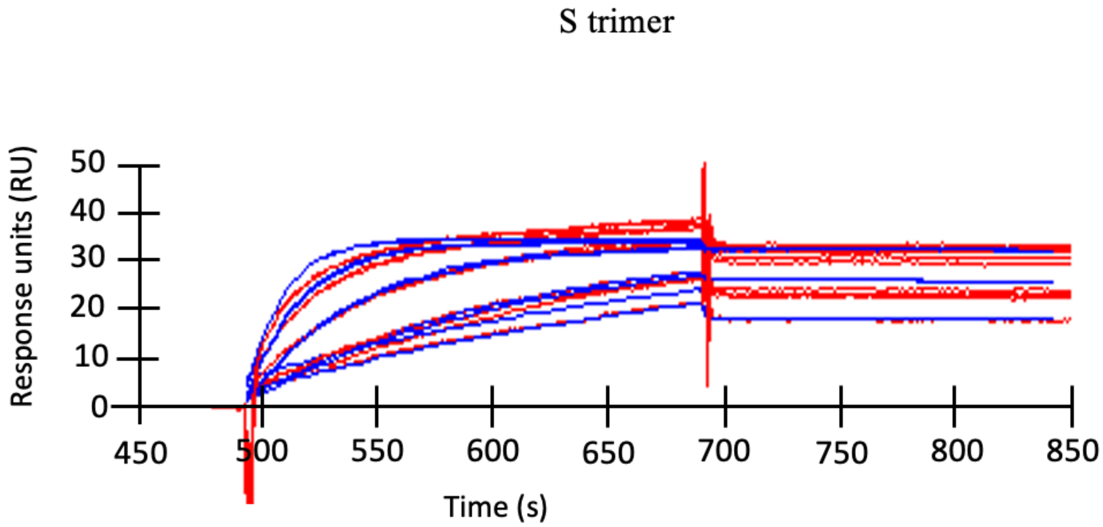

Figure S1: SPR analysis of bnAb pT1616 at 75, 50, 25, 10, and 5 nM to the immobilized SARS-CoV-2 RBD (A) or trimeric S protein (B). The blue lines represent a 1:1 kinetic model fit. The kinetic binding parameters are shown in Table S3.

Figure S2: Interaction of bnAb pT1616 with SARS-CoV-2 S trimer

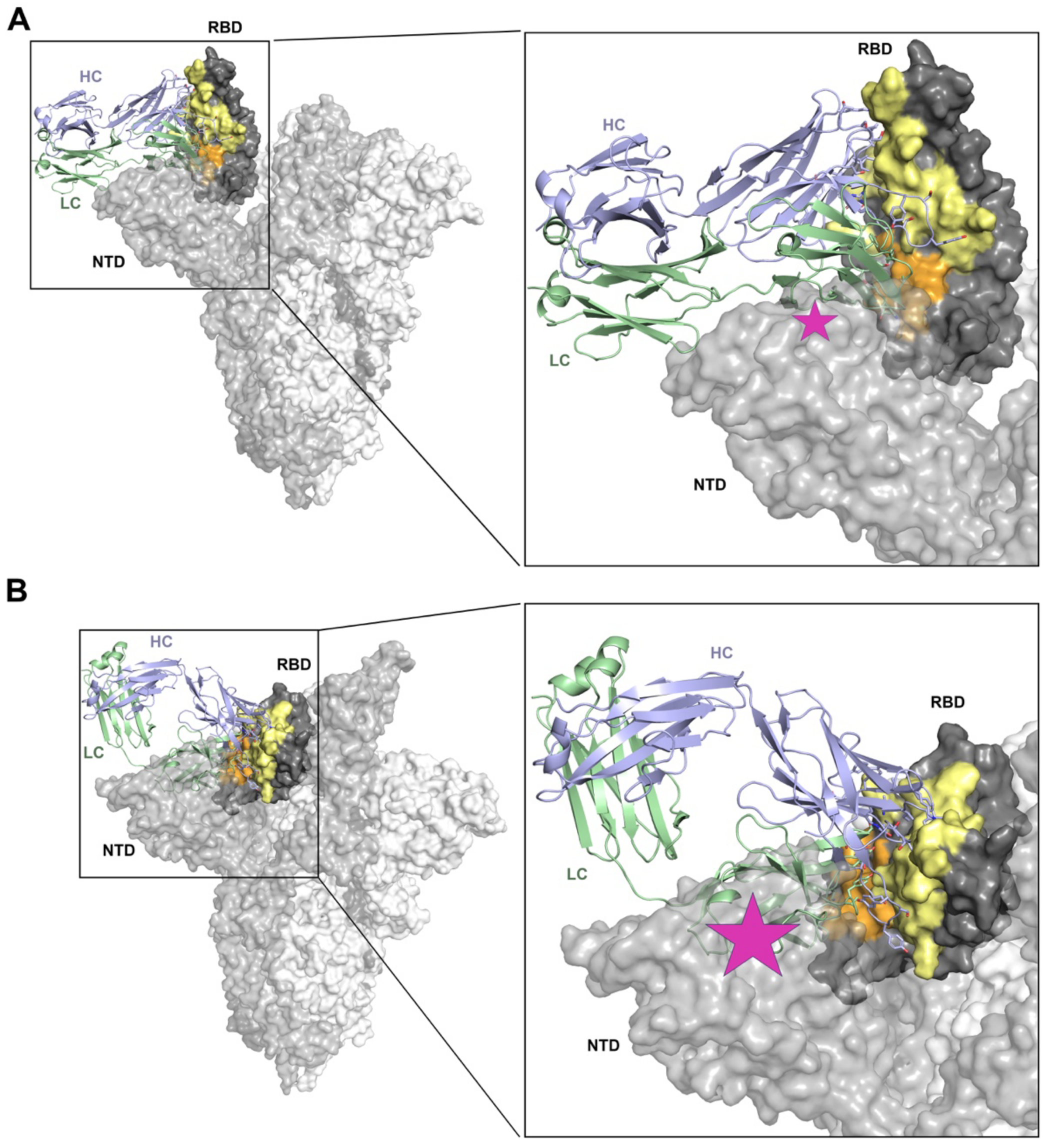

Figure S2: Superposition of the crystal structure of pT1616 in complex with the SARS-CoV-2 RBD to the S protein trimer (PDB 7WZ2) with an RBD in "up" (A) or "down" (B) conformation. The S trimer is shown in surface representation with individual protomers colored in different shades of gray. The pT1616 antibody is colored in light blue (HC) and dark green (LC). Coloured regions on the RBD represent residues that are within 3.5 Å from bnAb pT1616 heavy (yellow) and light chain (orange), respectively. Clashes with the juxtaposed NTD (represented by a pink asterisk) are minor in the RBD "up" conformation and more pronounced, when the pT1616 complex is superposed to an RBD in "down" conformation.

Plasmid name	Pango	WHO classification	Citation
pCAGGS-VSV-G			(1)
pCG1-SARS-2-SΔ18			(2)
pCG1-SARS-SΔ18			(3)
pCG1-WIV-1-SΔ18			(4)
pCG1-MERS-SΔ20			this publication
pCG1-GD-1-2019-SΔ18			this publication
pCG1-pCoV-GX-P5L-SΔ18			this publication
pCG1-BANAL-20-236-SΔ18			this publication
pCG1-SARS-2-SΔ18_mut5	B.1.1.7	Alpha	this publication
pCG1-SARS-2-SΔ18_mut6	B.1.351	Beta	this publication
pCG1-SARS-2-SΔ18 (B.1.1.28)	B.1.1.28	Gamma	(5)
pCG1-SARS-2-SΔ18_mut9	B.1.617.2	Delta	this publication
pCG1-SARS-2-SΔ18_mut7	B.1.427	Epsilon	this publication
pCG1-SARS-2-SΔ18_mut8	B.1.526	Iota	this publication
pCG1-SARS-2-SΔ18_mut10	C.37	Lambda	this publication
pCG1-SARS-2-SΔ18_mut11	B.1.621	Mu	this publication
pCG1-SARS-2-SΔ18 Omicron BA.1	B.1.1.529	Omicron BA.1	(6)
pCG1-SARS-2-SΔ18_mut13	B.1.1.529.2	Omicron BA.2	this publication
pCG1-SARS-2-SΔ18 Omicron BA.2.75.2		Omicron BA.2.75.2	(7)
pCDNA3.1_SARS2-Spike BA.4/5 Δ21	B.1.1.529.4 B.1.1.529.5	Omicron BA.4/5	(8)
pCG1-SARS-2-SΔ18 Omicron BA.4.6		Omicron BA.4.6	(9)
pCDNA3.1_SARS2-Spike BQ.1.1 Δ21		Omicron BQ.1.1	(10)
pCG1-SARS-2-SΔ18 Omicron XBB.1.5		Omicron XBB.1.5	(11)
pCG1-SARS-2SΔ18 Omicron BA.2.86		Omicron BA.2.86	(12)
pCG1-SARS-2SΔ18 Omicron EG.5.1		Omicron EG.5.1	(13)

Table S1. Betacoronavirus S proteins used for VSV pseudotype virus generation in this study.

S proteins	Origin	Genbank accession
SARS-CoV-2	Wuhan-hu-1	MN908947
CoVZC45	bat / China	MG772933.1
HKU3-1	bat / China	DQ022305
WIV-1	bat / China	KC881007
BM48-31	bat / Bulgaria	GU190215.1
MERS-CoV	human / Saudi Arabia	JX869059
SARS-CoV	human / China	AY278488
RsSHC014	bat / China	KC881005
RaTG13	bat / Yunnan-China	MN996532
GX-P5L	pangolin / China	MT040333.1
GD/1/2019	pangolin / China	MT799524.1
SARS-CoV-2 B1.1.529	human	UJN96880.1

Table S2. Betacoronavirus S proteins recombinantly expressed in this study.

	RBD			S trimer		
	**k_a ($M^{-1}s^{-1}$)**	**k_d (s^{-1})**	**K_D (M)**	**k_a ($M^{-1}s^{-1}$)**	**K_d (s^{-1})**	**K_D (M)**
**pT1616**	7.8×10^5	1.9×10^{-4}	**2.4×10^{-10}**	8.4×10^5	6.9×10^{-5}	**8.3×10^{-11}**

Table S3. Kinetic parameters of pT1616 binding to the SARS-CoV-2 RBD and trimeric S protein.

**Complex**	pT1616-RBD
**PDB ID**	8RRN
**Data collection and processing**	
Resolution range	44.3 - 3.11 (3.23 - 3.12)
Space group	C2
Unit cell	173.100 59.060 152.030<br>90 95.45 90
Total reflections	182606 (17429)
Unique reflections	27745 (2663)
Multiplicity	6.6 (6.5)
Completeness (%)	99.47 (97.58)
Mean I/sigma(I)	7.17 (1.02)
Wilson B-factor	97.24
R-merge	0.4093 (2.741)
R-meas	0.4464 (2.983)
R-pim	0.1754 (1.16)
CC1/2	0.967 (0.333)
CC*	0.991 (0.707)
**Refinement**	
Reflections used in refinement	27709 (2661)
Reflections used for R-free	1384 (133)
R-work	0.2432 (0.3688)
R-free	0.2913 (0.4171)
CC(work)	0.880 (0.521)
CC(free)	0.874 (0.447)
Antibody:RBD complexes/AU	2
Number of non-hydrogen atoms	9517
macromolecules	9517
ligands	0
solvent	0
Protein residues	1261
RMS(bonds)	0.005
RMS(angles)	0.75
**Validation**	
MolProbity score	1.83
All-atom clashscore	4.01
Ramachandran favored (%)	94.92
Ramachandran allowed (%)	6.08

Ramachandran outliers (%)	0.00
Rotamer outliers (%)	0.28
**B factors (Å²)**	
Average B-factor	108.92
macromolecules	108.92
ligands	-
solvent	-

Table S4. X-ray crystallography data collection, refinement and validation statistics.

Supplementary references

1. Brinkmann C, Hoffmann M, Lubke A, Nehlmeier I, Kramer-Kuhl A, Winkler M, Pohlmann S. 2017. The glycoprotein of vesicular stomatitis virus promotes release of virus-like particles from tetherin-positive cells. PLoS One 12:e0189073. 10.1371/journal.pone.0189073
2. Hoffmann M, Kleine-Weber H, Pöhlmann S. 2020. A Multibasic Cleavage Site in the Spike Protein of SARS-CoV-2 Is Essential for Infection of Human Lung Cells. Molecular Cell 78:779-784.e5. 10.1016/j.molcel.2020.04.022
3. Hoffmann M, Muller MA, Drexler JF, Glende J, Erdt M, Gutzkow T, Losemann C, Binger T, Deng H, Schwegmann-Wessels C, Esser KH, Drosten C, Herrler G. 2013. Differential sensitivity of bat cells to infection by enveloped RNA viruses: coronaviruses, paramyxoviruses, filoviruses, and influenza viruses. PLoS One 8:e72942. 10.1371/journal.pone.0072942
4. Vanshylla K, Fan C, Wunsch M, Poopalasingam N, Meijers M, Kreer C, Kleipass F, Ruchnewitz D, Ercanoglu MS, Gruell H, Munn F, Pohl K, Janicki H, Nolden T, Bartl S, Stein SC, Augustin M, Dewald F, Gieselmann L, Schommers P, Schulz TF, Sander LE, Koch M, Luksza M, Lassig M, Bjorkman PJ, Klein F. 2022. Discovery of ultrapotent broadly neutralizing antibodies from SARS-CoV-2 elite neutralizers. Cell Host Microbe 30:69-82 e10. 10.1016/j.chom.2021.12.010
5. Hoffmann M, Arora P, Groß R, Seidel A, Hörnich BF, Hahn AS, Krüger N, Graichen L, Hofmann-Winkler H, Kempf A, Winkler MS, Schulz S, Jäck H-M, Jahrsdörfer B, Schrezenmeier H, Müller M, Kleger A, Münch J, Pöhlmann S. 2021. SARS-CoV-2 variants B.1.351 and P.1 escape from neutralizing antibodies. Cell 184:2384-2393.e12. 10.1016/j.cell.2021.03.036
6. Arora P, Zhang L, Krüger N, Rocha C, Sidarovich A, Schulz S, Kempf A, Graichen L, Moldenhauer A-S, Cossmann A, Dopfer-Jablonka A, Behrens GMN, Jäck H-M, Pöhlmann S, Hoffmann M. 2022. SARS-CoV-2 Omicron sublineages show comparable cell entry but differential neutralization by therapeutic antibodies. Cell Host & Microbe 30:1103-1111.e6. 10.1016/j.chom.2022.04.017
7. Arora P, Kempf A, Nehlmeier I, Schulz SR, Jack HM, Pohlmann S, Hoffmann M. 2023. Omicron sublineage BQ.1.1 resistance to monoclonal antibodies. Lancet Infect Dis 23:22-23. 10.1016/S1473-3099(22)00733-2
8. Gruell H, Vanshylla K, Korenkov M, Tober-Lau P, Zehner M, Münn F, Janicki H, Augustin M, Schommers P, Sander LE, Kurth F, Kreer C, Klein F. 2022. SARS-CoV-2 Omicron sublineages exhibit distinct antibody escape patterns. Cell Host & Microbe 30:1231-1241.e6. 10.1016/j.chom.2022.07.002
9. Arora P, Zhang L, Nehlmeier I, Kempf A, Cossmann A, Dopfer-Jablonka A, Schulz SR, Jack HM, Behrens GMN, Pohlmann S, Hoffmann M. 2022. The effect of cilgavimab and neutralisation by vaccine-induced antibodies in emerging SARS-CoV-2 BA.4 and BA.5 sublineages. Lancet Infect Dis 22:1665-1666. 10.1016/S1473-3099(22)00693-4
10. Dewald F, Pirkl M, Paluschinski M, Kuhn J, Elsner C, Schulte B, Knufer J, Ahmadov E, Schlotz M, Oral G, Bernhard M, Michael M, Luxenburger M, Andree M, Hennies MT, Hafezi W, Muller MM, Kumpers P, Risse J, Kill C, Manegold RK, von Frantzki U,

Richter E, Emmert D, Monzon-Posadas WO, Graff I, Kogej M, Buning A, Baum M, Teipel F, Mochtarzadeh B, Wolff M, Gruell H, Di Cristanziano V, Burst V, Streeck H, Dittmer U, Ludwig S, Timm J, Klein F. 2023. Impaired humoral immunity to BQ.1.1 in convalescent and vaccinated patients. Nat Commun 14:2835. 10.1038/s41467-023-38127-y

11. Hoffmann M, Arora P, Nehlmeier I, Kempf A, Cossmann A, Schulz SR, Morillas Ramos G, Manthey LA, Jack HM, Behrens GMN, Pohlmann S. 2023. Profound neutralization evasion and augmented host cell entry are hallmarks of the fast-spreading SARS-CoV-2 lineage XBB.1.5. Cell Mol Immunol 20:419-422. 10.1038/s41423-023-00988-0
12. Zhang L, Kempf A, Nehlmeier I, Cossmann A, Richter A, Bdeir N, Graichen L, Moldenhauer AS, Dopfer-Jablonka A, Stankov MV, Simon-Loriere E, Schulz SR, Jack HM, Cicin-Sain L, Behrens GMN, Drosten C, Hoffmann M, Pohlmann S. 2024. SARS-CoV-2 BA.2.86 enters lung cells and evades neutralizing antibodies with high efficiency. Cell 187:596-608 e17. 10.1016/j.cell.2023.12.025
13. Zhang L, Kempf A, Nehlmeier I, Cossmann A, Dopfer-Jablonka A, Stankov MV, Schulz SR, Jack HM, Behrens GMN, Pohlmann S, Hoffmann M. 2023. Neutralisation sensitivity of SARS-CoV-2 lineages EG.5.1 and XBB.2.3. Lancet Infect Dis 23:e391-e392. 10.1016/S1473-3099(23)00547-9
